# Anthropometric Trajectories in Children Prior to Development of Inflammatory Bowel Disease

**DOI:** 10.1001/jamanetworkopen.2024.55158

**Published:** 2025-01-17

**Authors:** Maiara Brusco De Freitas, Gry Juul Poulsen, Tine Jess

**Affiliations:** 1 Center for Molecular Prediction of Inflammatory Bowel Disease, Department of Clinical Medicine, Aalborg University, Copenhagen, Denmark; 2 Department of Gastroenterology and Hepatology, Aalborg University Hospital, Aalborg, Denmark

## Abstract

**Importance:**

Poor nutrition and growth in childhood have short-term and long-term consequences, so understanding the timing of the onset of an impaired nutritional status is crucial for diagnosing and treating inflammatory bowel disease (IBD) at its earliest stage.

**Objective:**

To assess anthropometric trajectories before a pediatric diagnosis of IBD and growth recovery after diagnosis.

**Design, Setting, and Participants:**

This population-based cohort study included children born in Denmark from January 1, 1997, through December 31, 2015, with weight and length or height measurements at birth and at least 1 length or height and weight measurement at school age based on the Danish Medical Birth Register and the Danish National Child Health Register. Within this population, all individuals diagnosed with IBD at ages 5 to 17 years, according to the Danish National Patient Register, were identified. Data were analyzed from October 13, 2023, to April 17, 2024.

**Exposure:**

A pediatric diagnosis of IBD compared with the corresponding population without the disease.

**Main Outcomes and Measures:**

The outcome measures were *z* scores for length or height, weight, and body mass index (BMI [calculated as weight in kilograms divided by height in meters squared]) before and after pediatric IBD diagnosis compared with reference and sibling populations.

**Results:**

The final study population included 916 133 individuals (51.2% male) with a median of 3 pairs of length or height and weight measurements collected (IQR, 2-6 pairs). Of those, 1522 (median age, 14.3 years [IQR, 11.8-16.3 years]; 763 female [50.1%]) were diagnosed with IBD (851 [55.9%] with Crohn disease [CD] and 671 [44.1%] with ulcerative colitis [UC]). Compared with children without IBD, individuals with a later diagnosis of CD had declining anthropometric measures 3 years (weight: mean, −0.12 g [95% CI, −0.20 to −0.03 g]; BMI: mean, −0.13 [95% CI, −0.21 to −0.04]) and 1 year (length or height: mean, −0.20 cm [95% CI, −0.29 to −0.10 cm]) prior to diagnosis, whereas this was observed 1 year prior to a diagnosis of UC for weight (mean, −0.12 g [95% CI, −0.22 to −0.02 g]) and BMI (mean, −0.13 [95% CI, −0.23 to −0.03]). Deviating anthropometric patterns persisted after diagnosis, with the slowest recovery observed in children with CD.

**Conclusions and Relevance:**

The findings of this large-scale population-based cohort study of anthropometrics in children suggest impaired nutritional status as assessed by weight up to 3 years and by length or height 1 year before a diagnosis of CD and by weight up to 1 year before a diagnosis of UC. These findings emphasize that the onset of pediatric IBD may occur years prior to diagnosis, that growth recovery may first occur after diagnosis and treatment, and that frequent nutritional screenings may help ensure a healthy transition to adulthood.

## Key Points

**Question:**

What is the trajectory of anthropometric nutritional status prior to the development of inflammatory bowel disease (IBD) in childhood?

**Findings:**

In this cohort study, an unselected population dataset with consecutive anthropometric measurements of 916 133 children showed a decline in length or height, weight, and body mass index in those who later developed IBD, which persisted after diagnosis. The anthropometric deviations were more pronounced in children with Crohn disease, who showed reduced weight gain up to 3 years before diagnosis and reduced linear growth 1 year before diagnosis.

**Meaning:**

These results highlight the possible long preclinical phase of IBD in children, particularly among those with Crohn disease, and suggest the importance of frequent nutritional screenings to help ensure a healthy transition to adulthood.

## Teaser

This cohort study assesses the trajectory of anthropometric nutritional status in children before a diagnosis of inflammatory bowel disease (IBD) as well as growth recovery after diagnosis.

## Introduction

Immune-mediated inflammatory diseases such as inflammatory bowel disease (IBD) usually affect young adults.^1,2^ In 2017, there were 6.8 million cases of IBD worldwide, with more than 50 000 people affected in Denmark alone. The incidence of pediatric IBD in Europe is currently 15.3 per 100 000 person-years for Crohn disease (CD) and 14.8 per 100 000 person-years for ulcerative colitis (UC).^3,4,5,6^

Childhood-onset IBD can lead to impaired nutritional status, which may be the result of a complex interaction among diet, inflammation, hormones, and disease treatment, leading to malnutrition and impaired growth.^7,8,9^ Patients with CD appear to be more likely to experience linear growth retardation compared with those with UC.^9^

Early studies, with sample sizes ranging from 50 to 176 pediatric patients with IBD, suggested that growth impairment may already be present prior to IBD diagnosis^10^ or at least at the time of diagnosis^11,12,13^; may have a height-for-age *z* score at diagnosis varying between −0.5 and −2.0 SDs^14,15^; and may persist after diagnosis.^15^ However, data on prevalence, timing, and magnitude of growth impairment from unselected population-based studies with prediagnostic and postdiagnostic anthropometric measures on children with pediatric IBD are lacking. Understanding the timing of the onset of growth impairment may explain the timing of the onset of the disease, which is key for early diagnosis and treatment.

We therefore aimed to examine anthropometric trajectories, including *z* scores for length or height, weight, and body mass index (BMI [calculated as weight in kilograms divided by height in meters squared]), among children with IBD compared with those without IBD in a unique nationwide population-based cohort of Danish children with measurements from up to 10 years prior to IBD diagnosis to up to 3 years after IBD diagnosis.

## Methods

### Study Population

We used a cohort study design to assess anthropometrics in our study population. First, we identified all children born in Denmark from January 1, 1997, through December 31, 2015, through the Danish Medical Birth Register. By linkage to the Danish National Child Health Register (DNCHR), we excluded those with missing length and weight measurements at birth, implausible anthropometric measurements (cutoff points of 5 SDs) according to the Danish 2014 references,^16^ no anthropometric measurements before age 16.5 years, or no anthropometric measurements at school age (≥5.5 years). Ethical approval is not required for registry-based studies in Denmark. The study followed the rules and regulations defined by the Danish Data Protection Agency. Informed consent was not obtained or requested, as it is not necessary for registry-based studies in Denmark. The register data are protected by the Danish Act on Processing of Personal Data and are accessed through application to and approval from the Danish Data Protection Agency and the Danish Health Data Authority. This study followed the Strengthening the Reporting of Observational Studies in Epidemiology (STROBE) reporting guideline.

Within the study population, we identified all individuals diagnosed with IBD in childhood (ages 5 to 17 years) based on at least 2 outpatient or inpatient hospital contacts with IBD (*International Statistical Classification of Diseases, Tenth Revision* codes DK50 for CD and DK51 for UC) within 2 years in the Danish National Patient Register.^3,17^ If children had been recorded as having both CD and UC, they were classified as having CD. The date of IBD diagnosis was assigned as the first of the 2 contacts.

We further identified 1 full sibling (the sibling closest in age) without IBD-related hospital contacts to every child with IBD, if the child had a sibling, to address unmeasured family-level confounders. For the subgroup analysis, half-siblings and more than 1 sibling of the child with IBD were not included. Information on medications and family history of IBD was obtained from the Danish National Prescription and Patient Registries (eTable 1 in Supplement 1).

### Anthropometric Measurements

The DNCHR,^18^ established in April 2009, includes data from health care nurses and general practitioners. However, earlier records have been included by some municipalities, allowing us to access data prior to 2009.

In practice, anthropometric measurements are recorded at any age throughout childhood until the children leave school, but the majority of observations fall in infancy or around the scheduled visits during school years (eFigure 1 in Supplement 1). Given the inherently complex processes that govern anthropometric measurements of children in their first year of life, we chose to focus on measurements after children had reached 1 year of age.

For children with missing maternal height, we used mean height measured at the mother’s later births. After this imputation, maternal height was available for 62% of the population with IBD and 77% of the sibling population, while maternal BMI was available for 43% of the population with IBD and 60% of the sibling population.

### Statistical Analysis

Data were analyzed from October 13, 2023, to April 17, 2024. A Danish growth reference for height, weight, and BMI included the parameters necessary to compute *z* scores-for-age in 6-year intervals.^16^ However, deriving *z* scores for 6-year age intervals introduced considerable errors in the *z* scores, and for this reason, we only used the Danish growth reference to remove outliers, defined as individuals with height, weight, or BMI outside 5 SDs. Instead, we fitted generalized additive models for location, scale, and shape (GAMLSS) using the R package, version 4.3 (R Project for Statistical Computing). The GAMLSS model takes into account that mean, variance, and skewness change nonlinearly with age, and it was among the methods recommended by the World Health Organization.^19^

Since the DNCHR data include multiple observations on the final study population, we randomly drew a subsample consisting of 100 000 children and included 1 measurement per child. When picking the measurement for each child, we used a weighted sampling, up-weighting measurements from age intervals with few measurements. Next, GAMLSS models with Box-Cox power exponential distributions were fitted for all 3 growth measures separately for each sex, and *z* scores were computed for measurements from the IBD and sibling cohorts based on the fitted models (eFigure 2 in Supplement 1).

These steps transformed the data to normally distributed data and removed the effect of age and sex, which enabled us to model growth measures by years before and after IBD diagnosis. We included measurements from 10 years before to 3 years after IBD diagnosis and divided time in years. To assess whether the difference was associated with time of diagnosis, the overall population with IBD was also stratified into ages younger than 13 years and 13 years or older. Siblings of patients with IBD were given the date of diagnosis of their sibling as the index date. We modeled *z* scores by time in years before and after IBD diagnoses using a linear mixed-effects model with a random intercept for each child.

In a sensitivity analysis, we assessed the effect of using a random subsample by drawing 10 new samples and computing the mean *z* scores across the samples. However, due to the size of the register, the resampling was not associated with the results.

The statistical analysis was performed using R, version 4.3 (R Project for Statistical Computing). The results were presented as estimated *z* score means and 95% CIs. A 2-sided *P* value <.05 was considered significant.

## Results

**Figure 1.**
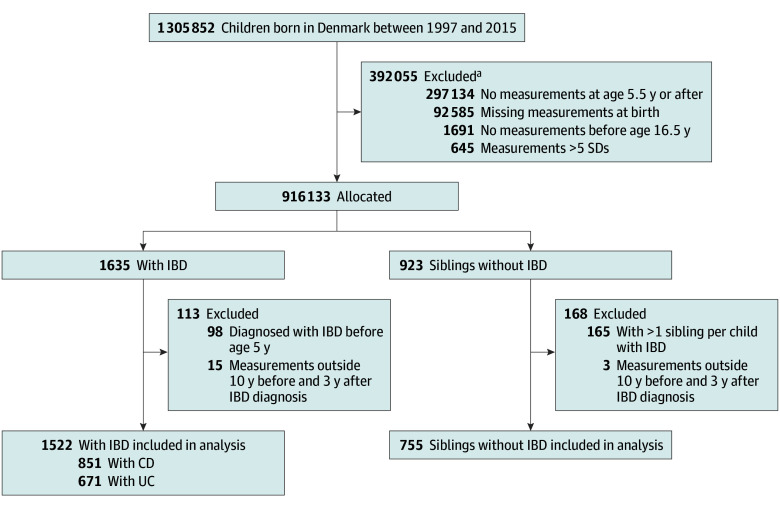
Flowchart of Study Participant Selection CD indicates Crohn disease; IBD, inflammatory bowel disease; UC, ulcerative colitis. ^a^Children could be excluded for more than 1 reason.

Among the 1 305 852 children born in Denmark between 1997 and 2015, the cohort consisted of 916 133 individuals (48.8% female and 51.2% male), with a median of 3 pairs of length or height and weight measurements (IQR, 2-6 pairs) collected (Figure 1 and Table 1). Among the children, the median birth weight was 3510 g (IQR, 3155-3870 g), and the median birth length or height was 52 cm (IQR, 50-53 cm). There were 1635 children with IBD with a diagnosis before age 18 years. Of these, 98 were diagnosed with IBD before age 5 years and were excluded. Of the remaining 1537 individuals, 1522 had measurements taken between 10 years before and 3 years after their IBD diagnosis (CD: 851 [55.9%]; UC: 671 [44.1%]).

**Table 1.** Characteristics According to Reference Individuals, Type of IBD, and Siblings Without IBD^a^

Characteristic	Individuals				Siblings without IBD (n = 755)
	Overall (N = 916 133)	With IBD (n = 1522)	With type of IBD		
			CD (n = 851)	UC (n = 671)	
**Children**					
Sex					
Female	447 288 (48.8)	763 (50.1)	411 (48.3)	352 (52.5)	368 (48.7)
Male	468 845 (51.2)	759 (49.9)	440 (51.7)	319 (47.5)	387 (51.3)
Year of birth^b^					
1997-2000	266 506 (29.1)	530 (34.8)	276 (32.4)	254 (37.9)	149 (19.7)
2001-2005	304 469 (33.2)	732 (48.1)	423 (49.7)	309 (46.1)	407 (53.9)
2006-2015	345 158 (37.7)	260 (17.1)	152 (17.9)	108 (16.1)	199 (26.4)
Measurements, median (IQR), No.	3 (2-6)	3 (2-4)	3 (2-4)	3 (2-4)	3 (2-4)
Birth weight, median (IQR), g	3510 (3155-3870)	3540 (3193-3890)	3560 (3200-3900)	3500 (3188-3875)	3520 (3185-3870)
Birth length or height, median (IQR), cm	52 (50-53)	52 (50-54)	52 (50-54)	52 (50-54)	52 (50-53)
Unknown	2097 (0.2)	<5	<5	<5	<5
Age at diagnosis, median (IQR), y	NA	14.3 (11.8-16.3)	14.1 (11.7-16.3)	14.5 (12.2-16.2)	12.6 (10.5-14.8)
Calendar year of diagnosis					
2003-2009	NA	33 (2.2)	15 (1.8)	18 (2.7)	NA
2010-2014	NA	327 (21.5)	179 (21.0)	148 (22.1)	NA
2015-2019	NA	751 (49.3)	426 (50.1)	325 (48.4)	NA
2020-2022	NA	411 (27.0)	231 (27.1)	180 (26.8)	NA
**Parents**					
Maternal height, median (IQR), cm	168 (164-172)	168 (164-172)	168 (164-172)	168 (164-172)	168 (164-172)
Unknown	264 826 (28.9)	594 (39.0)	320 (37.6)	274 (40.8)	181 (24.0)
Maternal BMI, median (IQR)	23.1 (20.9-26.4)	23.1 (21.0-26.0)	23.1 (21.0-26.0)	23.1 (20.7-26.3)	23.2 (21.1-26.5)
Unknown	411 614 (44.9)	892 (58.6)	491 (57.7)	401 (59.8)	312 (41.3)
Maternal IBD diagnosis	14 721 (1.6)	116 (7.6)	71 (8.3)	45 (6.7)	49 (6.5)
Paternal IBD diagnosis	12 466 (1.4)	97 (6.4)	51 (6.0)	46 (6.9)	55 (7.3)

Abbreviations: BMI, body mass index (calculated as weight in kilograms divided by height in meters squared); CD, Crohn disease; IBD, inflammatory bowel disease; NA, not applicable; UC, ulcerative colitis.

^a^ Data are presented as the No. (%) of individuals unless otherwise indicated.

^b^ The interval is combined with the previous interval to deidentify the 7 children with IBD.

The characteristics at birth of the 1522 children who later developed IBD (median age, 14.3 years [IQR, 11.8-16.3 years]; 763 female [50.1%] and 759 male [49.9%]) were similar to those of the overall sample and their 755 siblings without IBD. In the subgroup of children with IBD with known siblings without IBD (CD: 421 [55.8%]; UC: 334 [44.2%]), the median age at IBD diagnosis was 12.6 years (IQR, 10.5-14.8 years) (Table 1).

The distribution of the number of length or height and weight measurements performed in individuals who developed IBD in the period from 10 years before diagnosis to 3 years after diagnosis is presented in eTable 2 in Supplement 1. The height and the BMI of mothers were similar between groups, with an overall median height of 168 cm (IQR, 164-172 cm) and a median BMI of 23.1 (IQR, 20.9-26.4). Among the total number of parents of school-aged children included in the sample, 14 721 mothers (1.6%) and 12 466 fathers (1.4%) were diagnosed with IBD (Table 1).

### Anthropometric Measurements Before IBD Diagnosis

**Figure 2.**
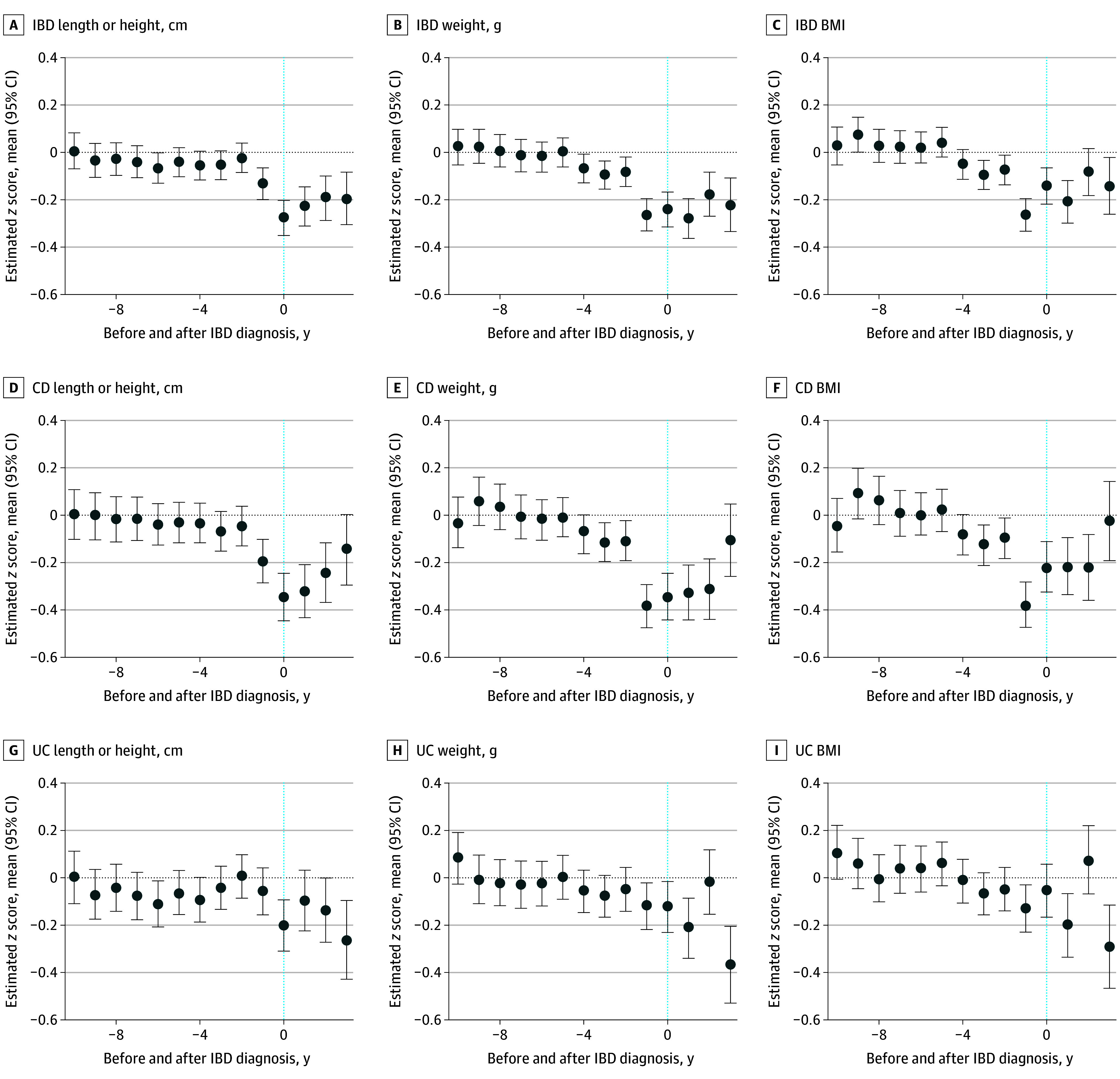
Estimated *z* Score Means of Length or Height, Weight, and Body Mass Index (BMI) Before and After Diagnosis of Inflammatory Bowel Disease (IBD) The dotted blue lines represent time 0 when patients with IBD, ulcerative colitis (UC), and Crohn disease (CD) were diagnosed. The *z* score mean (95% CI) of length or height, weight, and BMI (calculated as weight in kilograms divided by height in meters squared) before time 0 represents 10 years prior to diagnosis, and the area after time 0 represents 3 years following diagnosis.

We observed that 80 children (5.3%) diagnosed with IBD experienced temporary growth impairment (indicated by a length- or height-for-age of −2.0 or less SDs) at any point in time. In IBD overall, in which anthropometric measurements up to 10 years prior to the diagnosis were available, changes in weight were observed up to 4 years before (mean, −0.07 g [95% CI, −0.13 to −0.01 g]), reaching a mean −0.27 g (95% CI, −0.33 to −0.20 g) in the year before diagnosis, whereas impairment in linear growth was observed 1 year prior (mean, −0.14 cm [95% CI, −0.20 to −0.07 cm]), with significant worsening during the diagnostic period (mean, −0.28 cm [95% CI, −0.35 to −0.21 cm]) (Figure 2). Accordingly, alterations in BMI were observed from 3 years prior to diagnosis, with increasing impairment close to the time of diagnosis (mean, −0.27 [95% CI, −0.34 to −0.20]). Upon stratification of the data by age (<13 years and ≥13 years), it became evident that children who were diagnosed at an earlier age exhibited a more pronounced nutritional impairment compared with those diagnosed at a later age (eFigure 3 in Supplement 1).

These observations were primarily explained by anthropometric changes in children with CD, in which impairment in weight gain was observed 3 years before diagnosis (mean, −0.12 g [95% CI, −0.20 to −0.03 g]) with worsening in the last year before diagnosis (mean, −0.38 g [95% CI, −0.47 to −0.29 g]), and linear growth was a factor 1 year prior to diagnosis (mean, −0.20 cm [95% CI, −0.29 to −0.10 cm]) and during the diagnostic period (mean, −0.34 cm [95% CI, −0.44 to −0.24 cm]). Accordingly, BMI in children developing CD was significantly decreased 3 years prior to diagnosis (mean, −0.13 [95% CI, −0.21 to −0.04]), especially in the last year prior to diagnosis (mean, −0.38 [95% CI, −0.47 to −0.28]). In UC, we observed a slight deviation during the year prior to diagnosis in weight (mean, −0.12 g [95% CI, −0.22 to −0.02 g]) and in BMI (mean, −0.13 [95% CI, −0.23 to −0.03]), whereas for length or height, a slight impairment was present only in the year of diagnosis (mean, −0.20 cm [95% CI, −0.31 to −0.09 cm]).

### Anthropometric Measurements After IBD Diagnosis

When examining anthropometric measurements up to 3 years after a diagnosis of IBD in children, we observed that nutritional impairment persisted in the years following IBD diagnosis, particularly in those diagnosed at an earlier age. Children with CD approached the weight and length or height of children without IBD about 3 years after diagnosis, whereas children with UC seemed to recover within the first years after diagnosis but then showed significant deviation again in the third year (Figure 2). Recovery may be explained by treatment after diagnosis, which during the first year primarily included immunomodulators in children with CD (60%) and 5-aminosalicylic acid in children with UC (82.6%). However, systemic corticosteroids were used in 48.3% of children with CD and in 57.8% of children with UC in the first year, as were antitumor necrosis factor (anti-TNF) medications in 38.4% of children with CD and in 24.4% with UC (Table 2).

**Table 2.** Medications in the First Year After a Diagnosis of IBD in Children

Any medication	Children, No. (%)		
	With IBD disease (n = 1522)	With type of IBD	
		CD (n = 851)	UC (n = 671)
5-ASA	662 (43.5)	108 (12.7)	554 (82.6)
Systemic corticosteroids	799 (52.5)	411 (48.3)	388 (57.8)
Local corticosteroids	252 (16.6)	145 (17.0)	107 (15.9)
Immunomodulators	755 (49.6)	511 (60.0)	244 (36.4)
Anti-TNF	491 (32.3)	327 (38.4)	164 (24.4)
Non-TNF biologics	36 (2.4)	9 (1.1)	27 (4.0)

Abbreviations: 5-ASA, 5-aminosalicylic acid; CD, Crohn disease; IBD, inflammatory bowel disease; TNF, tumor necrosis factor; UC, ulcerative colitis.

### Siblings Without IBD

We assessed growth in siblings without IBD compared with individuals with IBD. We observed no deviation in anthropometric measurements in these children (eFigure 4 in Supplement 1). The complete estimated *z* score means and 95% CIs for length or height, weight, and BMI are shown in eTables 2 and 3 in Supplement 1.

## Discussion

This population-based cohort study of more than 900 000 children with consecutive anthropometric measurements revealed a decline in length or height, weight, and BMI in children who later developed IBD, particularly in those diagnosed at an earlier age, which persisted after diagnosis. The decline was most pronounced in children with CD, who had decreased weight *z* scores up to 3 years prior to diagnosis and decreased linear growth 1 year prior to diagnosis compared with children without IBD. Children diagnosed with UC showed significant anthropometric deviations at the time of diagnosis and impaired recovery in the long term despite promising recovery right after diagnosis. Treatment with corticosteroids and anti-TNF medications may be associated with the interpretation of early recovery.

Overall, our findings add to the mounting evidence that IBD has its onset several years prior to diagnosis^20,21,22,23^ and suggest that the outcome of the preclinical phase is associated with growth impairment in children, potentially due to impaired nutritional status. A comparison with previous studies is challenging, as such studies have been limited by the study size, a retrospective design, a lack of prediagnostic data, or a focus on the final height. The first study, to our knowledge, was published in 1988 and examined anthropometrics in 86 children and adolescents prior to their diagnosis of CD. The study revealed that 46% experienced reduced growth prior to the onset of symptoms, and 88% experienced reduced growth prior to diagnosis.^10^ A Canadian study published in 2020 with 1092 children aged 2 to 17 years^24^ found that the mean height *z* score was decreased in newly diagnosed children with CD (mean [SD], −0.30 [1.23]; 95% CI, −0.39 to −0.20) but not in children with an unclassified UC IBD (mean [SD], 0.11 [1.14]; 95% CI, −0.01 to 0.22). The longer-term effect of CD on nutritional and growth status was examined in 261 French children.^25^ Not only were the SDs for height (−0.38), weight (−1.14), and BMI (−1.37) significantly lower in patients at diagnosis than in the reference population, but deviations persisted after a median follow-up time of 73 months. This is similar to what we found for UC but not CD. The association of IBD with final height was examined in a Swedish National Patient Register study of 4201 individuals with childhood-onset IBD (CD: n = 1640; UC: n = 2201; and unclassified IBD: n = 360).^26^ The authors found that patients with pediatric IBD had a lower adult adjusted mean height difference compared with reference patients (−0.9 cm [95% CI, −1.1 to −0.7 cm]) and with their siblings without IBD (−0.8 cm [95% CI, −1.0 to −0.6 cm]). Additionally, patients with CD were slightly shorter than patients with UC (−1.3 cm vs −0.6 cm). The research also revealed that lower adult height was more frequently observed in patients with more severe disease progression (−1.9 cm [95% CI, −2.4 to −1.4 cm]). About 5.0% of individuals with CD and 4.3% of individuals with UC were classified with growth retardation compared with 2.5% of corresponding reference patients. Overall, this illustrates how growth and final height can be affected by IBD, and the present study highlights that these consequences may already be found years prior to IBD diagnosis. Moreover, growth can be regarded as a dynamic indicator of disease status, necessitating continuous monitoring.^27^

Impaired nutrition and growth in childhood can have short-term and long-term consequences if not treated effectively.^28,29^ The reduced weight in IBD may have multiple causes, such as decreased caloric intake due to the anorexic effects of proinflammatory mediators like interleukin (IL)-1β and TNF-α, malabsorption of food components, and/or early gastrointestinal symptoms, including early satiety, pain, and nausea.^30,31^ Reduced food intake may be caused by pain while eating, fear of diarrhea after meals, or poor tolerance of foods. Small-bowel involvement in IBD can further cause disaccharide intolerance, leading to shorter gut transit times, pain, and worsening of diarrhea.^32^ Impaired linear growth in individuals with IBD may be caused by factors associated with weight loss, as well as by mediators derived from the intestine.^33^ For example, TNF-α inhibits chondrocyte activity in growth plates, while IL-6 may directly lead to growth failure. Additionally, both can independently suppress levels of insulin-like growth factor-1, which is a critical mediator of the local actions of the growth hormone.^30,34,35,36^ Age at diagnosis, disease location, severity, treatment modality, and genetic polymorphisms are additional factors that may impact growth and weight impairment. In a cohort of Israeli children, the severity of disease as defined by steroid requirements, the use of immunosuppressives, and the cumulative period of hospitalization were found to be correlated with height and weight impairment.^37^ Some of the evidence has shown that an IL-6 gene polymorphism (−174 GG genotype) is associated with growth failure and more severe disease activity and that novel organic cation transporter 1/2 variants within the IBD5 haplotype may be determinants of disease susceptibility and worse nutritional status in early-onset IBD.^36,38^ Of note, we did not observe changes in anthropometrics for siblings without IBD to children with IBD, hence suggesting no family-derived confounders such as environment or common genetics.

It is also crucial to ascertain whether a medical intervention may reverse the condition subsequent to diagnosis. The results of this study corroborate previous findings that also observed an improvement in growth impairment after diagnosis, which may be a result of the treatment used.^39,40^ Although a study that evaluated the growth of 176 children with newly diagnosed CD (mean age, 10 years) with either mild (33%) or moderate or severe (67%) disease^15^ observed that exposure to corticosteroids for more than 6 months was associated with abnormal growth velocity after 1 year (odds ratio, 4.5 [95% CI, 2.2-9.6]), infliximab did not have a statistically significant effect. In contrast, studies conducted in the Swedish and Canadian populations found no association of the systemic use of corticosteroids with growth impairment.^9,26^ In our study, systemic corticosteroid was the second-most commonly used medication in the first year after diagnosis for CD and UC, which may provide an explanation for the apparent normalization of anthropometric measurements, particularly in UC, in the first year following diagnosis. However, deviations in anthropometric markers were observed in subsequent years.

### Strengths and Limitations

The primary strength of this study is the unique unselected population-based dataset with anthropometric measures on almost 1 million children. The DNCHR allowed us to identify all pediatric children with IBD in Denmark during the study period, in which we could derive growth patterns for up to 1 decade leading up to IBD diagnosis. By comparing results in children with IBD with full siblings without IBD, we were able to ascertain that potentially unmeasured family-level confounders were unlikely associated with the growth patterns in the IBD cohort. By using the full sample to create our own reference, we minimized the risk of introducing errors in the estimates, which may occur if using the general national reference since this is based on 6-year intervals, which may affect the final *z* score intervals.^19^

The study also has limitations to consider. Although the majority of measurements were taken at scheduled visits, in which all children were expected to be measured, a minor part of measurements was derived from additional visits, hence potentially introducing a minor imbalance to the longitudinal data. Although we could not estimate individual trajectories due to the limited number of observations for an individual child, we could still assess the effect of an IBD diagnosis on mean growth. Although we were able to report medical treatment in the early course of the disease, studying the direct effect of medications on growth would require a pharmacoepidemiologic study design.

## Conclusions

In this cohort study, nutritional impairment may have been present in children with CD at least 3 years prior to diagnosis and in children with UC in the year leading up to diagnosis. In children with UC, recovery seemed to occur within the first years after diagnosis, but that may reflect the outcomes of corticosteroids, and after the third year, anthropometric deviations were again evident. These results not only highlight the long preclinical phase of IBD, especially CD, but also demonstrate the significance of frequent nutritional screening and monitoring once children are diagnosed with IBD to restore nutritional status and provide a healthy transition to adulthood.

## Notes

Supplement 1. **eTable 1.** Number of Measurement and Unique Individuals by Time in the Inflammatory Bowel Disease (IBD) and Their IBD-Free Sibling’s Sample **eFigure 1.** Number of Observations by Age **eFigure 2.** Models Adjusted for All 3 Growth Measures Separately for Each Sex and *z* Scores **eTable 2.** Mean *z* Score per Year for Weight, Length or Height, and Body Mass Index Before and After Diagnosis for Inflammatory Bowel Disease, Crohn Disease, and Ulcerative Colitis **eFigure 3.** Estimated *z* Score Means With 95% CIs of Length or Height, Weight, and Body Mass Index (BMI) Before and After Diagnosis (Time 0) of Inflammatory Bowel Disease (IBD) Stratified by Age **eFigure 4.** Estimated *z* Score Means With 95% CIs of Length or Height, Weight, and Body Mass Index (BMI) Before and After Diagnosis (Time 0) of Siblings Without Inflammatory Bowel Disease (IBD) **eTable 3.** Mean *z* Score per Year for Weight, Length or Height, and Body Mass Index Before and After Diagnosis of Siblings Without Inflammatory Bowel Disease

Supplement 2.
Data Sharing Statement

## References

[1] Olén O, Askling J, Sachs MC, . Increased mortality of patients with childhood-onset inflammatory bowel diseases, compared with the general population. Gastroenterology. 2019;156(3):614-622. doi:10.1053/j.gastro.2018.10.028 30342031

[2] Bisgaard TH, Allin KH, Elmahdi R, Jess T. The bidirectional risk of inflammatory bowel disease and anxiety or depression: a systematic review and meta-analysis. Gen Hosp Psychiatry. 2023;83:109-116. doi:10.1016/j.genhosppsych.2023.05.002 37172544

[3] Agrawal M, Christensen HS, Bøgsted M, Colombel JF, Jess T, Allin KH. The rising burden of inflammatory bowel disease in Denmark over two decades: a nationwide cohort study. Gastroenterology. 2022;163(6):1547-1554.e5. doi:10.1053/j.gastro.2022.07.062 35952799 PMC9691534

[4] Alatab S, Sepanlou SG, Ikuta K, ; GBD 2017 Inflammatory Bowel Disease Collaborators. The global, regional, and national burden of inflammatory bowel disease in 195 countries and territories, 1990-2017: a systematic analysis for the Global Burden of Disease Study 2017. Lancet Gastroenterol Hepatol. 2020;5(1):17-30. doi:10.1016/S2468-1253(19)30333-4 31648971 PMC7026709

[5] Kaplan GG, Windsor JW. The four epidemiological stages in the global evolution of inflammatory bowel disease. Nat Rev Gastroenterol Hepatol. 2021;18(1):56-66. doi:10.1038/s41575-020-00360-x 33033392 PMC7542092

[6] Kuenzig ME, Fung SG, Marderfeld L, ; InsightScope Pediatric IBD Epidemiology Group. Twenty-first century trends in the global epidemiology of pediatric-onset inflammatory bowel disease: systematic review. Gastroenterology. 2022;162(4):1147-1159.e4. doi:10.1053/j.gastro.2021.12.282 34995526

[7] Shamir R, Phillip M, Levine A. Growth retardation in pediatric Crohn’s disease: pathogenesis and interventions. Inflamm Bowel Dis. 2007;13(5):620-628. doi:10.1002/ibd.20115 17262806

[8] Dipasquale V, Cucchiara S, Martinelli M, Miele E, Aloi M, Romano C. Challenges in paediatric inflammatory bowel diseases in the COVID-19 time. Dig Liver Dis. 2020;52(5):593-594. doi:10.1016/j.dld.2020.03.015 32276846 PMC7141464

[9] Duchatellier CF, Kumar R, Krupoves A, Braegger C, Herzog D, Amre DK. Steroid administration and growth impairment in children with Crohn’s disease. Inflamm Bowel Dis. 2016;22(2):355-363. doi:10.1097/MIB.0000000000000669 26752463

[10] Kanof ME, Lake AM, Bayless TM. Decreased height velocity in children and adolescents before the diagnosis of Crohn’s disease. Gastroenterology. 1988;95(6):1523-1527. doi:10.1016/S0016-5085(88)80072-6 3181677

[11] Kirschner BS. Growth and development in chronic inflammatory bowel disease. Acta Paediatr Scand Suppl. 1990;366:98-104. doi:10.1111/j.1651-2227.1990.tb11608.x 2206013

[12] Griffiths AM, Nguyen P, Smith C, MacMillan JH, Sherman PM. Growth and clinical course of children with Crohn’s disease. Gut. 1993;34(7):939-943. doi:10.1136/gut.34.7.939 8344582 PMC1374230

[13] Hildebrand H, Karlberg J, Kristiansson B. Longitudinal growth in children and adolescents with inflammatory bowel disease. J Pediatr Gastroenterol Nutr. 1994;18(2):165-173. doi:10.1097/00005176-199402000-00008 8014763

[14] Sawczenko A, Ballinger AB, Savage MO, Sanderson IR. Clinical features affecting final adult height in patients with pediatric-onset Crohn’s disease. Pediatrics. 2006;118(1):124-129. doi:10.1542/peds.2005-2931 16818557

[15] Pfefferkorn M, Burke G, Griffiths A, . Growth abnormalities persist in newly diagnosed children with Crohn disease despite current treatment paradigms. J Pediatr Gastroenterol Nutr. 2009;48(2):168-174. doi:10.1097/MPG.0b013e318175ca7f 19179878

[16] Tinggaard J, Aksglaede L, Sørensen K, . The 2014 Danish references from birth to 20 years for height, weight and body mass index. Acta Paediatr. 2014;103(2):214-224. doi:10.1111/apa.12468 24127859

[17] Albaek Jacobsen H, Jess T, Larsen L. Validity of inflammatory bowel disease diagnoses in the Danish National Patient Registry: a population-based study from the North Denmark region. Clin Epidemiol. 2022;14:1099-1109. doi:10.2147/CLEP.S378003 36226162 PMC9550174

[18] Andersen MP, Wiingreen R, Eroglu TE, . The Danish National Child Health Register. Clin Epidemiol. 2023;15:1087-1094. doi:10.2147/CLEP.S423587 38025840 PMC10656863

[19] Borghi E, de Onis M, Garza C, ; WHO Multicentre Growth Reference Study Group. Construction of the World Health Organization child growth standards: selection of methods for attained growth curves. Stat Med. 2006;25(2):247-265. doi:10.1002/sim.2227 16143968

[20] Vestergaard MV, Allin KH, Poulsen GJ, Lee JC, Jess T. Characterizing the pre-clinical phase of inflammatory bowel disease. Cell Rep Med. 2023;4(11):101263. doi:10.1016/j.xcrm.2023.101263 37939713 PMC10694632

[21] Torres J, Petralia F, Sato T, . Serum biomarkers identify patients who will develop inflammatory bowel diseases up to 5 years before diagnosis. Gastroenterology. 2020;159(1):96-104. doi:10.1053/j.gastro.2020.03.007 32165208

[22] Raygoza Garay JA, Turpin W, Lee SH, ; CCC GEM Project Research Consortium. Gut microbiome composition is associated with future onset of Crohn’s disease in healthy first-degree relatives. Gastroenterology. 2023;165(3):670-681. doi:10.1053/j.gastro.2023.05.032 37263307

[23] Rudbaek JJ, Agrawal M, Torres J, Mehandru S, Colombel JF, Jess T. Deciphering the different phases of preclinical inflammatory bowel disease. Nat Rev Gastroenterol Hepatol. 2024;21(2):86-100. doi:10.1038/s41575-023-00854-4 37950021 PMC11148654

[24] Dhaliwal J, Walters TD, Mack DR, . Phenotypic variation in paediatric inflammatory bowel disease by age: a multicentre prospective inception cohort study of the Canadian Children IBD Network. J Crohns Colitis. 2020;14(4):445-454. doi:10.1093/ecco-jcc/jjz106 31136648 PMC7242003

[25] Vasseur F, Gower-Rousseau C, Vernier-Massouille G, . Nutritional status and growth in pediatric Crohn’s disease: a population-based study. Am J Gastroenterol. 2010;105(8):1893-1900. doi:10.1038/ajg.2010.20 20145606

[26] Mouratidou N, Malmborg P, Sachs MC, . Adult height in patients with childhood-onset inflammatory bowel disease: a nationwide population-based cohort study. Aliment Pharmacol Ther. 2020;51(8):789-800. doi:10.1111/apt.15667 32133656

[27] Gupta N, Liu C, King E, ; ImproveCareNow Network. Continued statural growth in older adolescents and young adults with Crohn’s disease and ulcerative colitis beyond the time of expected growth plate closure. Inflamm Bowel Dis. 2020;26(12):1880-1889. doi:10.1093/ibd/izz334 31968095

[28] Dewey KG, Begum K. Long-term consequences of stunting in early life. Matern Child Nutr. 2011;7(suppl 3):5-18. doi:10.1111/j.1740-8709.2011.00349.x 21929633 PMC6860846

[29] Black RE, Allen LH, Bhutta ZA, ; Maternal and Child Undernutrition Study Group. Maternal and child undernutrition: global and regional exposures and health consequences. Lancet. 2008;371(9608):243-260. doi:10.1016/S0140-6736(07)61690-0 18207566

[30] Enomoto M, Pan HO, Kinoshita A, Yutani Y, Suzuki F, Takigawa M. Effects of tumor necrosis factor α on proliferation and expression of differentiated phenotypes in rabbit costal chondrocytes in culture. Calcif Tissue Int. 1990;47(3):145-151. doi:10.1007/BF02555979 2224589

[31] Man PS, Lawrence CB. Interleukin-1 mediates the anorexic and febrile actions of galanin-like peptide. Endocrinology. 2008;149(11):5791-5802. doi:10.1210/en.2008-0252 18617619 PMC3306896

[32] Moeeni V, Day AS. Impact of inflammatory bowel disease upon growth in children and adolescents. ISRN Pediatr. 2011;2011:365712. doi:10.5402/2011/365712 22389775 PMC3263571

[33] Ballinger A. Fundamental mechanisms of growth failure in inflammatory bowel disease. Horm Res. 2002;58(suppl 1):7-10. doi:10.1159/000064756 12373006

[34] De Benedetti F, Alonzi T, Moretta A, . Interleukin 6 causes growth impairment in transgenic mice through a decrease in insulin-like growth factor-I: a model for stunted growth in children with chronic inflammation. J Clin Invest. 1997;99(4):643-650. doi:10.1172/JCI119207 9045866 PMC507846

[35] Baker J, Liu JP, Robertson EJ, Efstratiadis A. Role of insulin-like growth factors in embryonic and postnatal growth. Cell. 1993;75(1):73-82. doi:10.1016/S0092-8674(05)80085-6 8402902

[36] Sawczenko A, Azooz O, Paraszczuk J, . Intestinal inflammation-induced growth retardation acts through IL-6 in rats and depends on the −174 IL-6 G/C polymorphism in children. Proc Natl Acad Sci U S A. 2005;102(37):13260-13265. doi:10.1073/pnas.0503589102 16150725 PMC1198995

[37] Wine E, Reif SS, Leshinsky-Silver E, . Pediatric Crohn’s disease and growth retardation: the role of genotype, phenotype, and disease severity. Pediatrics. 2004;114(5):1281-1286. doi:10.1542/peds.2004-0417 15520108

[38] Russell RK, Drummond HE, Nimmo ER, . Analysis of the influence of OCTN1/2 variants within the IBD5 locus on disease susceptibility and growth indices in early onset inflammatory bowel disease. Gut. 2006;55(8):1114-1123. doi:10.1136/gut.2005.082107 16469794 PMC1856267

[39] Walters TD, Kim MO, Denson LA, ; PRO-KIIDS Research Group. Increased effectiveness of early therapy with anti-tumor necrosis factor-α vs an immunomodulator in children with Crohn’s disease. Gastroenterology. 2014;146(2):383-391. doi:10.1053/j.gastro.2013.10.027 24162032

[40] Matar M, Shamir R, Lev-Zion R, . The effect of adalimumab treatment on linear growth in children with Crohn disease: a post-hoc analysis of the PAILOT randomized control trial. J Pediatr Gastroenterol Nutr. 2020;71(2):237-242. doi:10.1097/MPG.0000000000002728 32324651

