# Supplementary material for: Anthropometric Trajectories in Children Prior to Development of Inflammatory Bowel Disease
Source: JAMA Netw Open. 2025 Jan 17;8(1):e2455158. doi: 10.1001/jamanetworkopen.2024.55158 (PMC11742528; doi:10.1001/jamanetworkopen.2024.55158)
Supplement: Supplement 1. — eTable 1. Number of Measurement and Unique Individuals by Time in the Inflammatory Bowel Disease (IBD) and Their IBD-Free Sibling’s Sample eFigure 1. Number of Observations by Age eFigure 2. Models Adjusted for All 3 Growth Measures Separately for Each Sex and z Scores eTable 2. Mean z Score per Year for Weight, Length or Height, and Body Mass Index Before and After Diagnosis for Inflammatory Bowel Disease, Crohn Disease, and Ulcerative Colitis eFigure 3. Estimated z Score Means With 95% CIs of Length or Height, Weight, and Body Mass Index (BMI) Before and After Diagnosis (Time 0) of Inflammatory Bowel Disease (IBD) Stratified by Age eFigure 4. Estimated z Score Means With 95% CIs of Length or Height, Weight, and Body Mass Index (BMI) Before and After Diagnosis (Time 0) of Siblings Without Inflammatory Bowel Disease (IBD) eTable 3. Mean z Score per Year for Weight, Length or Height, and Body Mass Index Before and After Diagnosis of Siblings Without Inflammatory Bowel Disease [file jamanetwopen-e2455158-s001.pdf]

# Supplemental Online Content

Brusco De Freitas M, Poulsen GJ, Jess T. Antrhopometric trajectories in children prior to development of inflammatory bowel disease. *JAMA Netw Open*. 2025;8(1):e2455158. doi:10.1001/jamanetworkopen.2024.55158

**eTable 1.** Number of Measurement and Unique Individuals by Time in the Inflammatory Bowel Disease (IBD) and Their IBD-Free Sibling's Sample

**eFigure 1.** Number of Observations by Age

**eFigure 2.** Models Adjusted for All 3 Growth Measures Separately for Each Sex and *z* Scores

**eTable 2.** Mean *z* Score per Year for Weight, Length or Height, and Body Mass Index Before and After Diagnosis for Inflammatory Bowel Disease, Crohn Disease, and Ulcerative Colitis

**eFigure 3.** Estimated *z* Score Means With 95% CIs of Length or Height, Weight, and Body Mass Index (BMI) Before and After Diagnosis (Time 0) of Inflammatory Bowel Disease (IBD) Stratified by Age

**eFigure 4.** Estimated *z* Score Means With 95% CIs of Length or Height, Weight, and Body Mass Index (BMI) Before and After Diagnosis (Time 0) of Siblings Without Inflammatory Bowel Disease (IBD)

**eTable 3.** Mean *z* Score per Year for Weight, Length or Height, and Body Mass Index Before and After Diagnosis of Siblings Without Inflammatory Bowel Disease

This supplemental material has been provided by the authors to give readers additional information about their work.

© 2025 Brusco De Freitas M et al. *JAMA Network Open*.

**eTable 1.** Number of Measurement and Unique Individuals by Time in the Inflammatory Bowel Disease (IBD) and Their IBD-Free Sibling's Sample

| Time | Inflammatory bowel disease | IBD-free siblings |
|---|---|---|
| | measurements | measurements |
| -10 | 243 | 88 |
| -9 | 283 | 121 |
| -8 | 348 | 151 |
| -7 | 357 | 183 |
| -6 | 441 | 233 |
| -5 | 503 | 230 |
| -4 | 523 | 262 |
| -3 | 535 | 285 |
| -2 | 511 | 263 |
| -1 | 368 | 223 |
| 0 | 278 | 173 |
| 1 | 187 | 129 |
| 2 | 145 | 101 |
| 3 | 94 | 79 |

© 2025 Brusco De Freitas M et al. *JAMA Network Open*.

**eFigure 1**. Number of Observations by Age

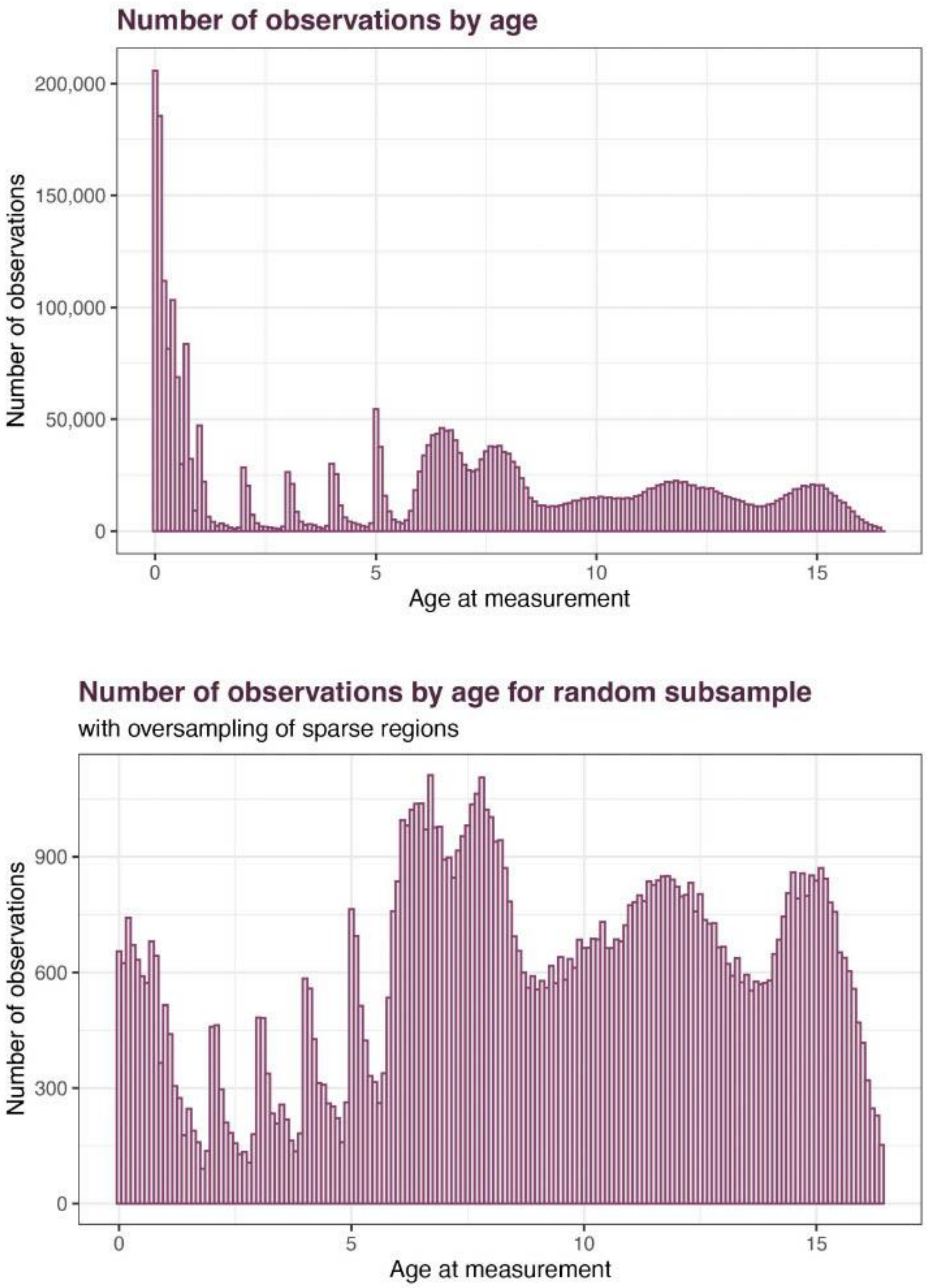


© 2025 Brusco De Freitas M et al. *JAMA Network Open*.

**eFigure 2.** Models Adjusted for All 3 Growth Measures Separately for Each Sex and *z* Scores

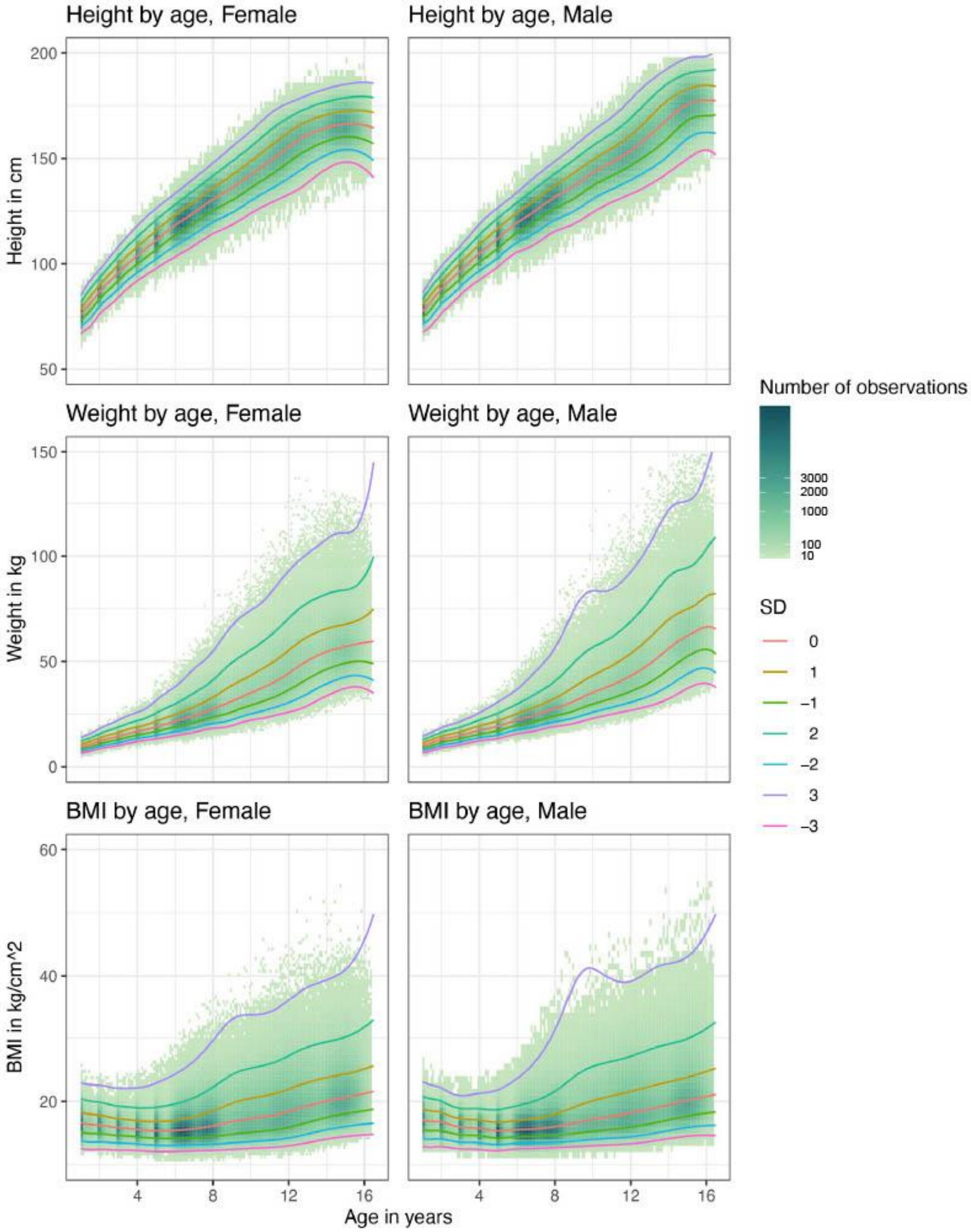


BMI: Body mass index. SD: Standard deviation.

© 2025 Brusco De Freitas M et al. *JAMA Network Open*.

**eTable 2.** Mean *z* Score per Year for Weight, Length or Height, and Body Mass Index Before and After Diagnosis for Inflammatory Bowel Disease, Crohn Disease, and Ulcerative Colitis

| **Years before and after diagnosis** | **Inflammatory bowel disease (95% CI)** | | **Crohn's disease (95% CI)** | | **Ulcerative colitis (95% CI)** | |
|---|---|---|---|---|---|---|
| **Length/height** | **Estimate** | **P value** | **Estimate** | **P value** | **Estimate** | **P value** |
| **-10** | 0·00 (-0·07, 0·08) | 0·944 | 0·00 (-0·10, 0·11) | 0·952 | 0·00 (-0·11, 0·11) | 0·960 |
| **-9** | -0·04 (-0·11, 0·04) | 0·317 | 0·00 (-0·10, 0·09) | 0·932 | -0·07 (-0·17, 0·03) | 0·191 |
| **-8** | -0·03 (-0·1, 0·04) | 0·376 | -0·02 (-0·11, 0·08) | 0·704 | -0·04 (-0·14, 0·06) | 0·400 |
| **-7** | -0·04 (-0·11, 0·03) | 0·225 | -0·02 (-0·11, 0·08) | 0·740 | -0·08 (-0·18, 0·02) | 0·141 |
| **-6** | -0·07 (-0·13, 0) | 0·039 | -0·04 (-0·12, 0·05) | 0·383 | -0·11 (-0·21, -0·01) | 0·028 |
| **-5** | -0·04 (-0·11, 0·02) | 0·164 | -0·03 (-0·12, 0·05) | 0·459 | -0·06 (-0·15, 0·03) | 0·193 |
| **-4** | -0·06 (-0·12, 0) | 0·067 | -0·03 (-0·12, 0·05) | 0·429 | -0·09 (-0·18, 0·00) | 0·054 |
| **-3** | -0·06 (-0·12, 0·01) | 0·076 | -0·07 (-0·15, 0·01) | 0·101 | -0·04 (-0·13, 0·05) | 0·387 |
| **-2** | -0·02 (-0·09, 0·04) | 0·452 | -0·05 (-0·13, 0·04) | 0·273 | 0·01 (-0·09, 0·10) | 0·882 |
| **-1** | -0·14 (-0·2, -0·07) | **0·000** | -0·20 (-0·29, -0·10) | **0·000** | -0·06 (-0·16, 0·04) | 0·273 |
| **0** | -0·28 (-0·35, -0·21) | **0·000** | -0·34 (-0·44, -0·24) | **0·000** | -0·2 (-0·31, -0·09) | **0·000** |
| **1** | -0·23 (-0·31, -0·14) | **0·000** | -0·32 (-0·43, -0·21) | **0·000** | -0·1 (-0·22, 0·03) | 0·146 |
| **2** | -0·2 (-0·29, -0·1) | **0·000** | -0·24 (-0·37, -0·12) | **0·000** | -0·13 (-0·27, 0·00) | 0·053 |
| **3** | -0·2 (-0·31, -0·08) | **0·001** | -0·15 (-0·29, 0·00) | 0·054 | -0·26 (-0·43, -0·1) | **0·002** |
| **Weight** | | | | | | |
| **-10** | 0·02 (-0·06, 0·10) | 0·595 | -0·03 (-0·14, 0·07) | 0·568 | 0·08 (-0·02, 0·19) | 0·131 |
| **-9** | 0·02 (-0·05, 0·10) | 0·503 | 0·06 (-0·04, 0·16) | 0·260 | 0·00 (-0·11, 0·1) | 0·928 |
| **-8** | 0·00 (-0·06, 0·07) | 0·914 | 0·03 (-0·06, 0·13) | 0·474 | -0·02 (-0·12, 0·08) | 0·678 |
| **-7** | -0·02 (-0·08, 0·05) | 0·639 | -0·01 (-0·1, 0·08) | 0·869 | -0·03 (-0·13, 0·07) | 0·586 |
| **-6** | -0·02 (-0·09, 0·04) | 0·505 | -0·02 (-0·11, 0·06) | 0·616 | -0·02 (-0·12, 0·07) | 0·634 |
| **-5** | 0·00 (-0·06, 0·06) | 0·958 | -0·01 (-0·09, 0·07) | 0·830 | 0·00 (-0·09, 0·09) | 0·941 |
| **-4** | -0·07 (-0·13, -0·01) | **0·025** | -0·08 (-0·16, 0·00) | 0·055 | -0·06 (-0·15, 0·03) | 0·223 |
| **-3** | -0·10 (-0·16, -0·04) | **0·002** | -0·12 (-0·20, -0·03) | **0·006** | -0·08 (-0·17, 0·01) | 0·090 |
| **-2** | -0·08 (-0·15, -0·02) | **0·008** | -0·11 (-0·19, -0·03) | **0·010** | -0·05 (-0·14, 0·04) | 0·301 |
| **-1** | -0·27 (-0·33, -0·20) | **0·000** | -0·38 (-0·47, -0·29) | **0·000** | -0·12 (-0·22, -0·02) | **0·019** |
| **0** | -0·24 (-0·32, -0·17) | **0·000** | -0·34 (-0·44, -0·24) | **0·000** | -0·12 (-0·23, -0·01) | **0·026** |
| **1** | -0·28 (-0·37, -0·20) | **0·000** | -0·33 (-0·44, -0·21) | **0·000** | -0·21 (-0·34, -0·08) | **0·001** |
| **2** | -0·18 (-0·27, -0·09) | **0·000** | -0·31 (-0·44, -0·18) | **0·000** | -0·02 (-0·15, 0·12) | 0·811 |
| **3** | -0·22 (-0·33, -0·11) | **0·000** | -0·11 (-0·26, 0·04) | 0·166 | -0·37 (-0·53, -0·20) | **0·000** |
| **Body mass index** | | | | | | |
| **-10** | 0·02 (-0·06, 0·10) | 0·562 | -0·04 (-0·16, 0·07) | 0·439 | 0·11 (-0·01, 0·22) | 0·061 |
| **-9** | 0·07 (0·00, 0·15) | 0·059 | 0·09 (-0·02, 0·19) | 0·103 | 0·06 (-0·04, 0·17) | 0·253 |
| **-8** | 0·03 (-0·04, 0·10) | 0·476 | 0·06 (-0·04, 0·16) | 0·223 | 0·00 (-0·10, 0·10) | 0·974 |
| **-7** | 0·02 (-0·05, 0·09) | 0·550 | 0·01 (-0·09, 0·10) | 0·869 | 0·04 (-0·06, 0·14) | 0·469 |
| **-6** | 0·02 (-0·05, 0·08) | 0·570 | 0·00 (-0·08, 0·09) | 0·941 | 0·04 (-0·06, 0·14) | 0·438 |
| **-5** | 0·04 (-0·02, 0·10) | 0·202 | 0·02 (-0·06, 0·11) | 0·614 | 0·06 (-0·03, 0·15) | 0·197 |
| **-4** | -0·05 (-0·11, 0·01) | 0·101 | -0·08 (-0·17, 0·00) | 0·057 | -0·01 (-0·10, 0·08) | 0·782 |
| **-3** | -0·10 (-0·16, -0·04) | **0·002** | -0·13 (-0·21, -0·04) | **0·004** | -0·06 (-0·15, 0·02) | 0·155 |
| **-2** | -0·08 (-0·14, -0·01) | **0·017** | -0·10 (-0·18, -0·01) | **0·027** | -0·05 (-0·14, 0·04) | 0·316 |
| **-1** | -0·27 (-0·34, -0·20) | **0·000** | -0·38 (-0·47, -0·28) | **0·000** | -0·13 (-0·23, -0·03) | **0·013** |
| **0** | -0·15 (-0·22, -0·07) | **0·000** | -0·22 (-0·32, -0·11) | **0·000** | -0·05 (-0·16, 0·06) | 0·350 |

© 2025 Brusco De Freitas M et al. *JAMA Network Open*.

| | | | | | | |
|---|---|---|---|---|---|---|
| **1** | -0·21 (-0·30, -0·12) | **0·000** | -0·22 (-0·34, -0·09) | **0·000** | -0·20 (-0·33, -0·07) | **0·004** |
| **2** | -0·08 (-0·18, 0·02) | 0·097 | -0·22 (-0·36, -0·08) | **0·002** | 0·08 (-0·07, 0·22) | 0·294 |
| **3** | -0·14 (-0·26, -0·02) | **0·021** | -0·02 (-0·19, 0·14) | 0·772 | -0·29 (-0·47, -0·11) | **0·001** |

Numbers in bold mean statistical difference in relation to the year of diagnosis.
95% confidence interval: 95% CI.

© 2025 Brusco De Freitas M et al. *JAMA Network Open*.

**eFigure 3.** Estimated *z* Score Means With 95% CIs of Length or Height, Weight, and Body Mass Index (BMI) Before and After Diagnosis (Time 0) of Inflammatory Bowel Disease (IBD) Stratified by Age

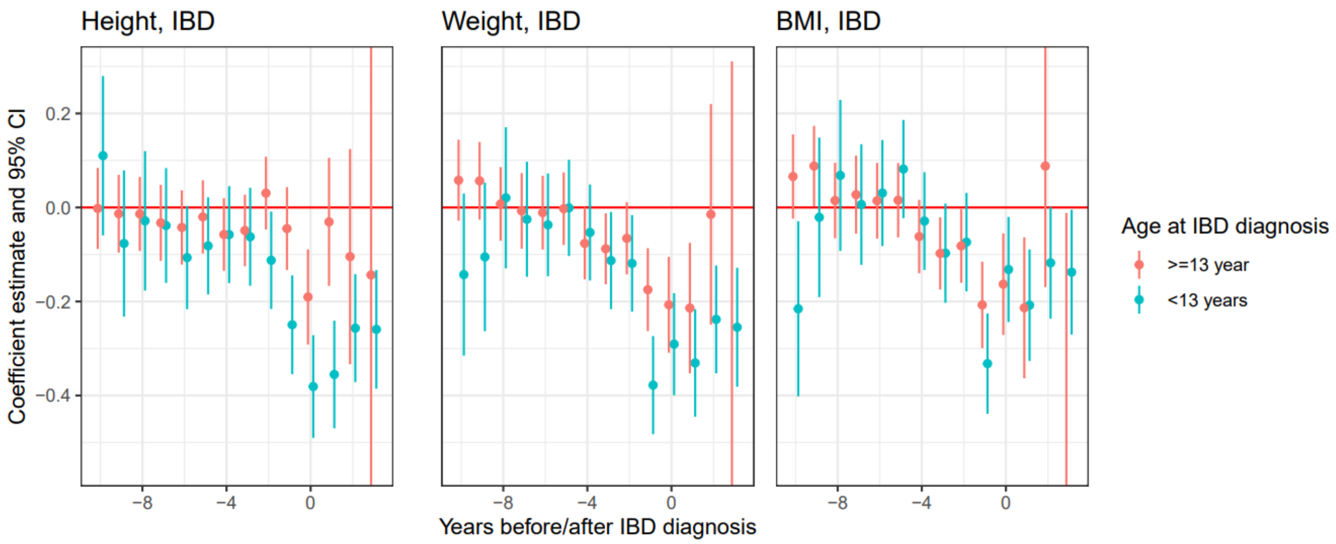


**eFigure 4.** Estimated *z* Score Means With 95% CIs of Length or Height, Weight, and Body Mass Index (BMI) Before and After Diagnosis (Time 0) of Siblings Without Inflammatory Bowel Disease (IBD)

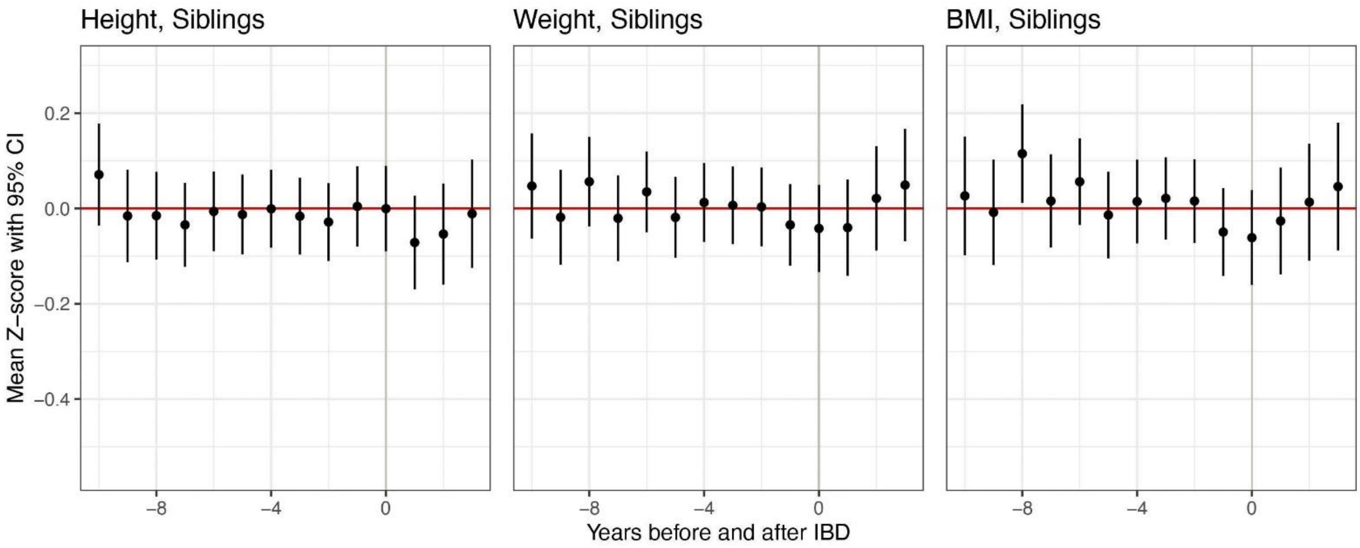


© 2025 Brusco De Freitas M et al. *JAMA Network Open*.

**eTable 3.** Mean *z* Score per Year for Weight, Length or Height, and Body Mass Index Before and After Diagnosis of Siblings Without Inflammatory Bowel Disease

| **Length/height** | **Estimate** | **P value** |
|---|---|---|
| **-10** | 0·07 (-0·04, 0·18) | 0·192 |
| **-9** | -0·02 (-0·11, 0·08) | 0·752 |
| **-8** | -0·02 (-0·11, 0·08) | 0·749 |
| **-7** | -0·03 (-0·12, 0·05) | 0·446 |
| **-6** | -0·01 (-0·09, 0·08) | 0·886 |
| **-5** | -0·01 (-0·10, 0·07) | 0·767 |
| **-4** | 0·00 (-0·08, 0·08) | 0·987 |
| **-3** | -0·02 (-0·10, 0·06) | 0·691 |
| **-2** | -0·03 (-0·11, 0·05) | 0·495 |
| **-1** | 0·00 (-0·08, 0·09) | 0·920 |
| **0** | 0·00 (-0·09, 0·09) | 0·994 |
| **1** | -0·07 (-0·17, 0·03) | 0·154 |
| **2** | -0·05 (-0·16, 0·05) | 0·320 |
| **3** | -0·01 (-0·12, 0·10) | 0·849 |
| **Weight** | | |
| **-10** | 0·05 (-0·06, 0·16) | 0·402 |
| **-9** | -0·02 (-0·12, 0·08) | 0·716 |
| **-8** | 0·06 (-0·04, 0·15) | 0·242 |
| **-7** | -0·02 (-0·11, 0·07) | 0·654 |
| **-6** | 0·03 (-0·05, 0·12) | 0·420 |
| **-5** | -0·02 (-0·1, 0·07) | 0·665 |
| **-4** | 0·01 (-0·07, 0·10) | 0·766 |
| **-3** | 0·01 (-0·07, 0·09) | 0·874 |
| **-2** | 0·00 (-0·08, 0·09) | 0·937 |
| **-1** | -0·03 (-0·12, 0·05) | 0·431 |
| **0** | -0·04 (-0·13, 0·05) | 0·369 |
| **1** | -0·04 (-0·14, 0·06) | 0·435 |
| **2** | 0·02 (-0·09, 0·13) | 0·704 |
| **3** | 0·05 (-0·07, 0·17) | 0·414 |
| **Body mass index** | | |
| **-10** | 0·03 (-0·10, 0·15) | 0·677 |
| **-9** | -0·01 (-0·12, 0·10) | 0·885 |
| **-8** | 0·11 (0·01, 0·22) | 0·029 |
| **-7** | 0·02 (-0·08, 0·11) | 0·752 |
| **-6** | 0·06 (-0·03, 0·15) | 0·226 |
| **-5** | -0·01 (-0·10, 0·08) | 0·767 |
| **-4** | 0·01 (-0·07, 0·10) | 0·747 |
| **-3** | 0·02 (-0·06, 0·11) | 0·628 |
| **-2** | 0·02 (-0·07, 0·10) | 0·727 |
| **-1** | -0·05 (-0·14, 0·04) | 0·292 |
| **0** | -0·06 (-0·16, 0·04) | 0·228 |
| **1** | -0·03 (-0·14, 0·09) | 0·646 |
| **2** | 0·01 (-0·11, 0·14) | 0·832 |
| **3** | 0·05 (-0·09, 0·18) | 0·502 |

95% confidence interval: 95% CI.

© 2025 Brusco De Freitas M et al. *JAMA Network Open*.
