# Supplementary material for: Anthropometric Trajectories in Children Prior to Development of Inflammatory Bowel Disease
Source: JAMA Netw Open. 2025 Jan 17;8(1):e2455158. doi: 10.1001/jamanetworkopen.2024.55158 (PMC11742528; doi:10.1001/jamanetworkopen.2024.55158)
Supplement: Supplement 2. — Data Sharing Statement [file jamanetwopen-e2455158-s002.pdf]

Data Sharing Statement

De Freitas. Anthropometric Trajectories in Children Prior to Development of Inflammatory Bowel Disease. *JAMA Netw Open*. Published January 17, 2025.
doi:10.1001/jamanetworkopen.2024.55158

Data
Data available: No

Additional Information
Explanation for why data not available: The sources for the outcome are national registry data which, cannot be made available to the public according to European law, but which researchers can apply for access to from the relevant health authorities.
